# The Joint Effects of Some Beverages Intake and Smoking on Chronic Obstructive Pulmonary Disease in Korean Adults: Data Analysis of the Korea National Health and Nutrition Examination Survey (KNHANES), 2008–2015

**DOI:** 10.3390/ijerph17072611

**Published:** 2020-04-10

**Authors:** Ji Eun Min, Da-An Huh, Kyong Whan Moon

**Affiliations:** 1 School of Health and Environmental Science, Korea University, 145 Anam-ro, Seongbuk-gu, Seoul 02841, Korea; adela32@korea.ac.kr; 2 Department of Health Science, Korea University, 145 Anam-ro, Seongbuk-gu, Seoul 02841, Korea; black1388@korea.ac.kr

**Keywords:** soda, coffee, green tea, smoking, lung function, chronic obstructive pulmonary disease, interaction, Korean adults

## Abstract

Some beverages and smoking cause an inflammatory response in the lungs and airways in a similar way, ultimately affecting chronic obstructive pulmonary disease (COPD) occurrence. Using a nationally representative health survey database, this study investigates the individual and joint effects of consumption of different beverages and smoking on COPD. This study is a cross-sectional analysis of 15,961 Korean adults in the Korea National Health and Nutritional Examination Survey of 2008–2015. COPD was defined as forced expiratory volume in 1 s (FEV_1_) divided by forced vital capacity (FVC) <0.70. We used multiple linear and logistic regression models to examine the association of beverage consumption and smoking with an FEV_1_/FVC ratio and odds ratio (OR) for COPD. The mean FEV_1_/FVC ratio decreased with increasing soda intake (*p* = 0.016), coffee intake (*p* = 0.031), and smoking status; however, the mean FEV_1_/FVC ratio increased with increasing green tea intake frequency (*p* = 0.029). When soda intake increased to 10 times/month, the OR of having COPD increased to 1.04 times (95% CI: 1.01, 1.07). The positive joint effect of soda intake and smoking on COPD was marginally significant (*p* = 0.058). We found that soda intake, coffee intake, and smoking increased airflow limitation while green tea intake decreased it. In addition, soda intake and smoking had a positive joint effect on COPD in the Korean population.

## 1. Introduction

Chronic obstructive pulmonary disease (COPD) is characterized by chronic lung inflammation, causing airway and lung parenchymal damage and leading to irreversible airflow restriction [1]. According to the Global Initiative for Chronic Obstructive Lung Disease, COPD is now the fourth leading cause of death worldwide, and it will become the third leading cause by 2020 [2]. The dominant risk factor for COPD is smoking, and about 15%–20% of smokers have COPD [3].

Smokers tend to consume high amounts of beverages such as soda, coffee, and green tea because of increased thirst after smoking [4,5]. Like smoking, these beverages also have an effect on pulmonary function. A study conducted in Australia suggested that soda intake increases the prevalence of asthma [6], and a different study showed that coffee intake increases the risk of lung cancer [7]. Additionally, the consumption of green tea has been shown to be associated with an increase in pulmonary function [8].

Previous studies have shown evidence of the individual effects of consumption of different beverages and of smoking on lung function; however, only a few studies have investigated the simultaneous exposure to smoking and beverage consumption on COPD [6,7]. Because some beverages have been shown to have a similar mechanism as smoking on lung function, their co-exposure could cause greater effect than individual exposure. For example, reactive oxidant substances produced by soda intake and smoking could jointly affect pulmonary inflammation resulting in COPD [9]. Therefore, the joint effects of these factors on lung function need to be addressed.

Using data from a nationwide representative health survey database, this study investigates the individual and joint effects of consumption of some beverages and of smoking on COPD.

## 2. Materials and Methods

### 2.1. Study Population

The Korea National Health and Nutrition Examination Survey (KNHANES)^7^, conducted periodically by the Korea Centers for Disease Control and Prevention (KCDC) since 1998, is the surveillance system for the general Korean population [10]. This survey is implemented to assess the health and nutrition status of South Koreans and monitor health-related risk factors and prevalence of diseases. The data in KNHANES consist of a health interview, health examination, and nutrition survey [11]. Before carrying out the survey, the ethical approval for the study protocol was provided by each participant. The institutional review board (IPB) approval codes from 2008 to 2014 are as follows: 2008-04EXP-01-C, 2009-01CON-03-2C, 2010-02CON-21-C, 2011-02CON-06-C, 2012-01EXP-01-2C, 2013-07CON-03-4C, and 2013-12EXP-03-5C. Since 2015, the KNHANES has been carried out without IRB review in accordance with the research conducted directly by the government for public welfare according to the bioethics law of Korea. We used secondary data from these for the epidemiology study.

**Figure 1:**
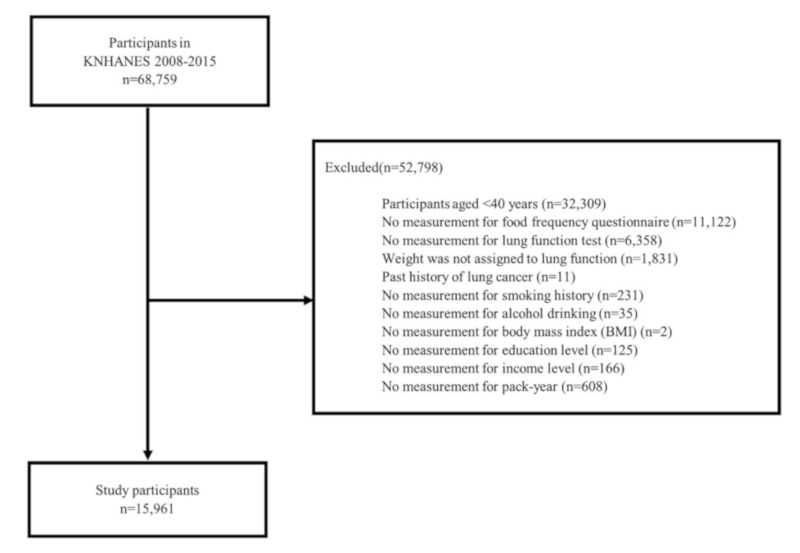
Study population (KNHAENS, Korea National Health and Nutrition Examination Survey, 2008–2015)**.**

This study examines data from 68,759 people in the KNHANES database of 2008–2015 (Figure 1). We excluded participants less than 40 years old (*n* = 32,309) because only participants ≥40 years were tested for lung function. Those who did not respond to the food frequency questionnaire (FFQ) (*n* = 11,122), those who did not perform spirometry tests (*n* = 6358), and those without weighting (*n* = 1831) were excluded. Participants who had a past medical history of lung cancer (*n* = 11) and those who did not have a measurement of smoking history (*n* = 231) were also excluded. We also excluded participants who did not have cofounding variable data: alcohol drinking (*n* = 35), body mass index (BMI; *n* = 2), education level (*n* = 125), monthly income (*n* = 166), and pack-year (*n* = 608). In total, 15,961 participants were selected for the study.

### 2.2. Beverage Intake

The consumption of soda, coffee, and green tea was assessed using data obtained from the food frequency included in the nutrition survey. A trained interviewer visited homes and conducted face-to-face interviews [12]. Beverages intake frequency was divided into nine categories: 3 times/day, 2 times/day, 1 time/day, 5–6 times/week, 2–4 times/week, 1 time/week, 2–3 times/month, and 1 time/month. These frequencies were thought to have little impact difference on lung function and had very few participants in each group. Therefore, we re-categorized the frequency of beverage intake into the following four groups: never (reference), ≤4 times/week, 5–7 times/week, and >7 times/week. The analysis was also performed with continuous variables by converting the previously-used nine categories into how many times the participants drink the given beverages per month.

### 2.3. Cigarette Smoking

Smoking was assessed by the self-administered questionnaire in the mobile examination center included in the health interview [11]. Study participants were divided into non-smokers, past-smokers, or current-smokers according to smoking status. Pack-years were calculated by multiplying the average number of cigarette packs per day by total years of smoking [13].

### 2.4. Definition of COPD

In the KNHANES database, the pulmonary function test was conducted on participants aged over 40 by using spirometry. Spirometry testing was performed by experts according to the recommendation of the American Thoracic Society/European Respiratory Society criteria for standardizing pulmonary function tests PETs [14]. COPD was defined as a forced expiratory volume in 1 s (FEV_1_) divided by forced vital capacity (FVC) <0.70 [2].

### 2.5. Covariates

We used demographic and lung function-related variables as potential confounders. The variables that we considered were sex, age, monthly income, education level, drinking status, and body mass index (BMI). Smoking status was used as a covariate when observing the effects of beverage intake on lung function, and beverage intake was used as a covariate when observing the effects of smoking on lung function. Data on monthly income were available as quartiles in each survey year, and education level was classified as <high school (reference), high school, or >high school. Alcohol consumption was classified as non-drinker, past-drinker, and current-drinker, and BMI was calculated as weight (kg)/height (m) squared.

### 2.6. Statistical Analysis

The KNHANES used the stratified multistage probability sampling design and sample weight the participants sample to represent the general population of Korea. We used an integrated weight value through the 2008–2015 KNHANES dataset and applied the KNHANES analysis tutorial for statistical analysis (KCDC 2014).

We used the Student *t*-test and the Wald F-test to evaluate the differences in the arithmetic mean between the groups. The Rao–Scott chi-square test was used to estimate the effects of variables on COPD prevalence.

Multiple linear regression analysis was used to evaluate the association of beverage intake frequency and smoking status with lung function. We also conducted a logistic regression analysis to estimate the odds ratio (OR) for COPD. We constructed a single model adjusted for sex, age, monthly income, education level, drinking status, and BMI in both linear and logistic regression analysis.

The joint effects of beverage intake and smoking on COPD were examined after adjusting for all covariates. We divided each beverage intake and smoking variable into low and high groups. Beverage intake was divided into ≤ 1 time/week (low) and, > 1 time/week (high), and smoking was divided into ≤20 pack-years (low) and, >20 pack-years (high). Then, we used the combinations of these categorical variables and classified them into the following four groups: low beverage intake and low pack-years (reference), low beverage intake and high pack-years, high beverage intake and low pack-years, and high beverage intake and high pack-years [15]. According to the recommendation of Knol and Vander-Weele, we calculated the additive scale (relative excess risk due to interaction, RERI) and the multiplicative scale (the ratio of ORs) [16]. We computed 95% confidence interval (CI) to RERI, following the standard delta method based on a Taylor Series expansion [17].

All statistical analyses were performed using SPSS version 23.0, and the statistical significance level for the two-sided test was set as 0.05.

### 2.7. Sensitivity Analysis

We conducted sensitivity analyses using another definition of COPD, FEV_1_/FVC < lower limit of normal (LLN). These sensitivity analyses were performed because the fixed ratio criterion of FEV_1_/FVC <0.70 for diagnosing COPD may be associated with overdiagnosis, particularly in the elderly [18]. The LLN was defined as the FEV_1_/FVC ratio corresponding to z score = −1.96 in normal population excluding current-smokers and asthma patients in each following age groups: 40–59, 60–69, and ≥70. COPD patients were defined as FEV1/FVC < 0.8010, 0.7605, and 0.7370 in 40–59, 60–69, and ≥70 age groups, respectively.

## 3. Result

### 3.1. Participants’ Characteristics

#### 3.1.1. According to the Frequency of Beverages Intake and the Smoking Status

Table 1 shows significant differences in the frequency of beverage intake and smoking status by study participants’ characteristics. The weighted arithmetic means (AM) and 95% CI of soda, coffee, and green tea were 16.3 (15.4, 17.1), 30.7 (30.0, 31.6), and 5.9 (5.6, 6.2) times/month, respectively. When categorizing the total population according to smoking status, the number and weighted percentage of never, past, and current -smokers were 10,190 (58.0%), 3,441 (24.1%), and 2330 (17.9%), respectively. The frequency of all beverage intake was higher for men than for women (*p* < 0.001). The proportion of non-smokers was higher for women, while the proportions of past-smokers and current-smokers were higher for men (*p* < 0.001). The frequency of beverage intake decreased with age (*p* < 0.001), similar to the rate of current -smokers (*p* < 0.001).

#### 3.1.2. According to the FEV_1_/FVC and COPD

Table 2 shows differences in FEV1/FVC and COPD prevalence by the study participants’ characteristics. Among the total 15,961 subjects, the weighted AM and 95% CI of FEV_1_/FVC was 0.78 (0.78, 0.79), and 1737 (10.5%) were patients with COPD. COPD prevalence tended to decrease as the intake of soda or green tea increased (both *p* < 0.001), and COPD prevalence increased with increased smoking and coffee consumption (smoking status: *p* < 0.001, coffee intake frequency: *p* = 0.009).

### 3.2. The Effects of Beverages Intake and Smoking Status on FEV_1_/FVC

Table 3 presents multiple linear regression analysis results to show the association of beverage intake and smoking with FEV_1_/FVC. In the crude model, as soda intake increased by 10 times a month, mean FEV_1_/FVC increased by 0.0011 (95% CI: 0.0006, 0.0016); however, after adjusting for all covariates, as soda intake increased by 10 times a month, mean FEV_1_/FVC decreased by 0.0008 (95% CI: −0.0012, −0.0003). In the fully adjusted model, as coffee intake increased by 10 times a month, mean FEV_1_/FVC decreased by 0.0006 (95% CI: −0.0010, −0.0001), and as green tea intake increased by 10 times a month, mean FEV_1_/FVC increased by 0.0009 (95% CI: 0.0001, 0.0017). When compared with non-smokers, current-smokers showed a greater negative correlation with FEV_1_/FVC (*β* = −0.0222; 95% CI: −0.0273, −0.0171).

### 3.3. ORs for COPD According to Beverages Intake and Smoking Status

Table 4 shows the multiple logistic linear analysis results of ORs for COPD according to the frequency of beverage consumption and smoking status. After adjusting for all covariates, as soda intake increased by 10 times a month, COPD prevalence increased by 1.04 (95% CI: 1.01, 1.07) times. Using non-smokers as a reference, COPD prevalence of past-smokers and current-smokers increased to 1.78 (95% CI: 1.42, 2.24) times and to 2.58 (95% CI: 2.06, 3.24) times, respectively. As coffee intake increased, COPD prevalence tended to increase to 1.01 (95% CI: 0.99, 1.03), and as green tea intake increased, COPD prevalence tended to decrease to 0.98 (95% CI: 0.93, 1.03), but both were not significant. In a sensitivity analysis, similar results have been observed. The results of it are presented in Table S1.

### 3.4. The Joint Effects of Beverages Intake and Smoking

Table 5, Table 6 and Table 7 respectively show the joint effects of soda, coffee, and green tea intake with smoking on COPD under simultaneous exposure. For soda, the OR for participants in both high groups when compared with the reference group was 3.00 (95% CI: 2.32, 3.89), for participants with a high pack-year only was 2.04 (95% CI: 1.68, 2.48), and for participants with high soda intake was 1.26 (95% CI: 1.02, 1.55). The RERI, which is an estimate of the joint effect on the additive scale of soda intake and smoking status, was 0.71 (95% CI: −0.02, 1.44). The observed additive effect was greater than the sum of the effects of soda intake and smoking alone. There was a positive interaction on the additive scale, and the result approached statistical significance (*p* = 0.058). Table 6 shows the joint effect of coffee intake and smoking, and Table 7 shows the joint effect of green tea intake and smoking. A positive interaction between coffee intake and smoking and a negative interaction between green tea and smoking were noted, although statistically insignificant on both additive and multiplicative scales.

## 4. Discussion

This study illustrates the individual and joint effects of the frequency of soda, coffee, or green tea consumption and smoking status on COPD using a representative sample of the Korean population in 2008–2015 KNHANES. For soda and coffee, airflow limitation increased with increasing intake frequency, similar to the airflow limitation increase seen in the smokers’ lungs. On the contrary, higher green tea intake frequency was associated with less airflow limitation. In addition, we have shown a joint effect of co-exposure to soda and smoking on COPD. We found that the OR for COPD was greater in the group simultaneously exposed to soda and smoking when compared with the sum OR of the high soda group and high smoking group.

We controlled demographic variables that may act as a potential confounder. Age is one of the major risk factors for COPD, and the risk for COPD increases with age [19]. COPD prevalence also depends on sex because of hormonal and lifestyle differences between males and females [20]. The findings that BMI affects pulmonary function exist [21,22]. Although the mechanism of these findings is not yet clear, respiratory muscle weakness, gas exchange disorder, and immune response inhibition are some potential mechanisms that have been suggested [23]. Monthly income and education level are surrogates for social economic status (SES), and SES is associated with chronic diseases [24]. In addition, studies have shown that continuous drinking causes airway inflammation and aggravates lung function [25]. We, therefore, controlled these variables in statistical analysis, even though they are not direct risk factors for COPD.

Interestingly, in Table 4, under the crude model, COPD prevalence decreased as the frequency of soda intake increased; however, after adjusting for all covariates, the prevalence increased as the frequency of soda intake increased. The average frequency of soda intake (95% CI) based on the characteristics of the study participants decreased to 19.2 (95% CI: 18.1, 20.2) times/month, 11.4 (95% CI: 10.3, 12.5) times/month and 1.0 (95% CI: 0.8, 1.3) times/month at age 40–59, 60–69, and ≥70, respectively. According to the American lung association, lung function declines gradually as we age because of muscle weakness in the diaphragm and because lung tissue that helps to keep the airways open loses elasticity. Due to these pieces of evidence, in the crude model, the majority of younger people belong to groups with a high level of soda intake, so it can be assumed that the higher the frequency of soda intake, the lower the COPD prevalence.

The findings of this study show that co-exposure of soda and smoking has a joint effect on COPD since both factors affect pulmonary function with an inflammatory response. Experimental evidence suggests that foods that promote oxidative stress and inflammation affect the pathogenesis of COPD since COPD is associated with inflammation [26]. Soda contains high amounts of sugar and sugar consumption increases the sensitivity of allergic airway inflammatory response [6]. Surfactant protein D (SP-D), a molecule in charge of the innate immune system of the lungs, limits the sensitivity of airway inflammatory disease by interacting with cellular components [27]. Sugar damages the immune defense system of SP-D and increases the susceptibility of airway inflammation [28]. In an animal experiment, sugar-fed mice had twice as much airway inflammation as water-fed mice [28]. Cigarette smoke contains high concentrations of oxidants that can cause an inflammatory response in the lung and airway [9]. In addition, cigarette smoke induces the release of neutrophil chemoattractant interleukin-8 in human bronchial epithelial cells, causing lung inflammation [29]. In the bronchial biopsies of central airways, smokers showed chronic inflammatory changes as the number of lung inflammatory cells increased [30].

The interaction between coffee intake and smoking was not significant, possibly because coffee intake affects COPD prevalence via a different mechanism to smoking. Most of the studies on caffeine provide evidence for a mechanism that affects the lungs with the spread of adenocarcinoma cells via some enzyme reactions. A study has shown that caffeine affects COPD and bronchial cancer, and it increases the risk or neonatal apnea in newborn babies [31]. Caffeine is a phosphodiesterase (PDE) inhibitor. In an animal experiment using hamsters, the PDE inhibitor provoked pulmonary adenocarcinoma of Clara cell lineage to hamsters [32]. In an experiment using human lung adenocarcinoma cells, caffeine also caused diffusion of pulmonary adenocarcinoma cells and small airway epithelial cells by activating protein kinase A (PKA), cyclic adenosine monophosphate (AMP) response element-binding protein (CREB), and extracellular signal-regulated kinases 1/2 (ERK1/2) [33].

The mechanism by which green tea has a protective effect on pulmonary function is not thought to have any interaction with smoking, which has a negative effect on pulmonary function. Catechin, an extract of green tea, has an antioxidant effect via an indirect mechanism (reactive oxygen species (ROS) scavengers, metal ion chelators) and via a direct mechanism (antioxidant enzyme inducers, pro-oxidant enzyme inhibitors, and stress-related signaling pathway suppressors) [34]. Characterized by this antioxidative activity, catechins are effective in preventing pathologies mediated by oxidative stress [35]. Catechin can also reduce inflammation in the lung tissue [8]. In an experiment, the mice given green tea decreased interstitial hemorrhaging and cellular infiltration in the lung tissues when compared with the control group [36].

This study has several strengths. First, this is the first study to investigate the interaction of co-exposure of soda, coffee, or green tea consumption and smoking on COPD in the Korean population. Several studies on the correlation between beverage consumption and smoking exist [4,5], and both factors affect pulmonary function via a similar mechanism. Therefore, we wanted to investigate the interaction of these factors. Soda and coffee intakes were expected to have a positive interaction with smoking because they are known to degrade lung function, while green tea was expected to have a negative interaction with smoking because it has been shown to improve lung function. When simultaneously exposed to soda and smoking, a significant positive joint effect on COPD was observed. Our study suggests that this is because both factors have similar mechanisms on lung function. Second, we focused on COPD, unlike most prior studies that investigated lung cancer among lung-related diseases [37,38]. COPD is a severe public health problem that increases socioeconomic burdens and mortality [39]. According to the Korean Health Insurance Review and Assessment Service database, the total cost of COPD-related medications in Korea rose 33.1% over 5 years [40]. Hong et al. said that constant monitoring and prevention are needed because of the substantial socioeconomic burden of COPD [41]. Third, unlike previous studies that investigated the effects of only one type of beverage on COPD [4,8], this study considered three different beverages; soda, coffee, and green tea, simultaneously. Since these three beverages are known to affect COPD prevalence via separate mechanisms, more significant results were obtained by controlling each beverage as confounders in the statistical analysis. Finally, the 2008–2015 KNHANES data used in this study represent the general population in Korea; therefore, the above results can be generalized.

The limitations of the present study should also be considered. First, KNHANES is a cross-sectional study and so we cannot guarantee the causality between beverage intake, smoking, and COPD. However, given the side-effects of soda, coffee, and smoking on health, and the benefits of green tea on health, a reverse relation between the intake of these beverages and smoking and COPD cannot be deduced logically. Second, FFQ of KNHANES was used to measure the frequency of beverage intake via interview, relying on the participants’ memories and likely leading to memory decay bias. Third, there are some factors that affect pulmonary function that could not be controlled in our study. There are cases where lung function seems to be improved by medicine, although actual lung function is bad, and other cases where lung function seems to be worsened by occupational exposure, although actual lung function is good. No study on the effect of nutrient intake on lung function has shown a protective effect. According to the Associate of the Royal Institute of Chemistry (ARIC), n-3 polyunsaturated fatty acids and dietary fiber have a positive effect on pulmonary function. In addition, we could not control the environmental factors such as air pollutants that are major risk factors determining lung function. Particulate meter 2.5 (PM 2.5) can cause asthma and COPD by activating inflammatory-associated cells and inducing oxidative stress [42]. For these reasons, the true value of lung function may have been overestimated or underestimated. Follow up studies on COPD should consider the effects of these factors. Finally, the survey on the frequency of beverage intake obtained from FFQ is data gathered over the past year. Therefore, it is difficult to explain the change in pulmonary function by the frequency of beverage intake.

## 5. Conclusions

In conclusion, this study provides evidence that soda, coffee, and smoking increase airflow limitation, while green tea decreases it. In addition, soda intake and smoking individually had a significant correlation with the different severity of COPD. Also, the joint effects of soda intake and smoking were significant when exposed simultaneously. The tendency of interaction with co-exposure to coffee or green tea intake and smoking was observed to an extent somewhat, but neither an additive scale nor a multiplicative scale was statistically significant. These results suggest that further studies are needed to investigate the interactions of the intake of these beverages and smoking in the general population.

## Figures and Tables

**Table 1:** Baseline characteristics of study participants according to the frequency of beverages intake and smoking status ^1^.

Variables	Overall	Frequency of Beverage Intake (Times/Month), AM ^2^ (95% CI ^3^)						Smoking Status, *n* (%) ^4^			
		Soda	*p*-Value	Coffee	*p*-Value	Green Tea	*p*-Value	Never	Past-Smoker	Current-Smoker	*p*-Value
Total	15,961 (100)	16.3 (15.4, 17.1)		30.7 (30.0, 31.6)		5.9 (5.6, 6.2)		10,190 (58.0)	3441 (24.1)	2330 (17.9)	
Sex			<0.001		<0.001		<0.001				<0.001
Male	6160 (45.6)	18.3 (17.2, 19.5)		35.8 (34.6, 37.1)		6.9 (6.4, 7.4)		1066 (16.8)	3111 (48.4)	2489 (34.9)	
Female	9801 (54.4)	14.5 (13.7, 15.4)		26.5 (25.6, 27.4)		5.0 (4.7, 5.3)		9124 (92.6)	330 (3.7)	347 (3.7)	
Age (years)			<0.001		<0.001		<0.001				<0.001
40–59	10,796 (74.6)	19.2 (18.1, 20.2)		31.8 (30.8, 32.9)		6.5 (6.2, 6.9)		7047 (57.3)	2042 (22.8)	1707 (19.9)	
60–69	3649 (16.5)	11.4 (10.3, 12.5)		26.4 (25.1, 27.8)		4.1 (3.7, 4.6)		2273 (60.4)	946 (27.7)	430 (11.9)	
≥70	1516 (8.9)	1.0 (0.8, 1.3)		29.5 (27.7, 31.4)		3.5 (2.8, 4.2)		870 (59.2)	453 (28.0)	193 (12.8)	
BMI (kg/m^2^)			0.116		<0.001		<0.001				<0.001
<25	10,146 (62.6)	15.8 (14.9, 16.7)		29.6 (28.6, 30.6)		5.3 (5.0, 5.6)		6599 (60.0)	2060 (22.4)	1487 (17.6)	
25–30	5230 (33.5)	17.0 (15.8, 18.3)		32.8 (31.6, 34.1)		6.9 (6.4, 7.5)		3167 (53.4)	1288 (27.8)	775 (18.8)	
≥30	585 (4.0)	17.2 (14.1, 20.2)		31.5 (28.0, 35.0)		5.9 (4.7, 7.1)		424 (64.7)	93 (19.4)	68 (15.9)	
Education level			<0.001		0.008		<0.001				<0.001
<High school	6731 (37.5)	10.3 (9.4, 11.1)		29.4 (28.3, 30.5)		4.2 (3.8, 4.5)		4666 (64.9)	1208 (19.8)	857 (15.3)	
High school	5226 (35.0)	19.3 (18.0, 20.7)		32.0 (30.6, 33.4)		6.0 (5.5, 6.5)		3269 (56.3)	1128 (24.4)	829 (19.3)	
>High school	4004 (27.6)	20.5 (18.9, 22.1)		31.0 (29.5, 32.6)		8.0 (7.4, 8.7)		2255 (50.8)	1105 (29.5)	644 (19.7)	
Monthly income			<0.001		<0.001		<0.001				0.002
First quartile	2815 (16.2)	9.1 (8.0, 10.2)		27.9 (26.4, 29.5)		4.1 (3.4, 4.7)		1791 (60.2)	577 (21.5)	447 (18.3)	
Second quartile	3956 (25.1)	15.6 (14.3, 17.0)		31.3 (29.8, 32.8)		5.0 (4.5, 5.5)		2529 (57.7)	818 (23.1)	609 (19.2)	
Third quartile	4168 (27.4)	17.6 (16.2, 19.0)		32.3 (30.7, 33.8)		6.1 (5.6, 6.7)		2624 (56.1)	940 (25.1)	604 (18.8)	
Fourth quartile	5022 (31.3)	19.3 (17.8, 20.9)		30.4 (29.0, 31.9)		7.3 (6.7, 7.8)		3246 (58.7)	1106 (25.3)	670 (16.0)	
Drinking status			<0.001		<0.001		<0.001				<0.001
Never	2473 (13.5)	10.2 (9.0, 11.5)		23.7 (22.1, 25.3)		4.6 (4.0, 5.2)		2225 (87.9)	130 (6.4)	118 (5.6)	
Past-drinker	2480 (14.8)	15.7 (14.1, 17.3)		26.4 (24.8, 28.0)		4.8 (4.1, 5.5)		1715 (65.4)	546 (24.7)	219 (9.9)	
Current-drinker	11,008 (71.7)	17.5 (16.5, 18.5)		33.0 (31.9, 34.0)		6.3 (6.0, 6.7)		6250 (50.8)	2765 (27.3)	1993 (21.9)	

^1.^ Student *t*-test and Wald F-test were used to evaluate the differences in the arithmetic mean between the groups. Rao–Scott chi-square test was used to compare the baseline characteristics across the categorical variables. ^2^ AM, arithmetic mean; ^3^ CI, confidence interval; ^4^ Except the number of participants, all values are weighted.

**Table 2:** Baseline characteristics of study participants according to the forced expiratory volume in 1 s (FEV1)/forced vital capacity (FVC) and chronic obstructive pulmonary disease (COPD) ^1^.

Variables	FEV_1_/FVC (%)		COPD, n (%) ^2^		
	AM (95% CI ^3^)	*p*-Value	Normal	Case	*p*-Value
Total	0.783 (0.781, 0.785)		14,224 (89.5)	1737 (10.5)	
Sex		<0.001			<0.001
Male	0.763 (0.761, 0.766)		4938 (83.3)	1222 (16.7)	
Female	0.799 (0.798, 0.801)		9286 (94.7)	515 (5.3)	
Age (years)		<0.001			<0.001
40–59	0.796 (0.795, 0.798)		10,181 (93.9)	615 (6.1)	
60–69	0.754 (0.751, 0.758)		2999 (81.2)	650 (18.8)	
≥70	0.727 (0.720, 0.733)		1044 (68.1)	472 (31.9)	
BMI (kg/m^2^)		<0.001			<0.001
<25	0.779 (0.777, 0.781)		8921 (88.3)	1225 (11.7)	
25–30	0.787 (0.785, 0.790)		4750 (91.1)	480 (8.9)	
≥30	0.803 (0.797, 0.809)		553 (94.6)	32 (5.4)	
Education		<0.001			<0.001
<High school	0.767 (0.764, 0.769)		5741 (84.2)	990 (15.8)	
High school	0.790 (0.788, 0.792)		4767 (91.9)	459 (8.1)	
> High school	0.796 (0.794, 0.798)		3716 (93.7)	288 (6.3)	
Monthly income		<0.001			<0.001
First quartile	0.756 (0.751, 0.761)		2272 (79.5)	543 (20.5)	
Second quartile	0.783 (0.781, 0.786)		3512 (89.9)	444 (10.1)	
Third quartile	0.789 (0.787, 0.792)		3805 (91.4)	363 (8.6)	
Fourth quartile	0.791 (0.789, 0.793)		4635 (92.7)	387 (7.3)	
Drinking status		0.008			0.137
Never	0.782 (0.777, 0.786)		2232 (89.3)	241 (10.7)	
Past-drinker	0.778 (0.774, 0.782)		2175 (88.0)	305 (12.0)	
Current-drinker	0.784 (0.782, 0.786)		9817 (89.8)	1191 (10.2)	
Smoking status		<0.001			<0.001
Never	0.798 (0.797, 0.800)		9624 (94.6)	556 (5.4)	
Past-smoker	0.763 (0.759, 0.766)		2771 (82.9)	670 (17.1)	
Current-smoker	0.761 (0.757, 0.765)		1829 (82.1)	501 (17.9)	
Soda intake frequency		<0.001			<0.001
Never	0.778 (0.776, 0.780)		6805 (87.8)	967 (12.2)	
≤4 times/week	0.785 (0.782, 0.787)		4103 (90.1)	490 (9.9)	
5–7 times/week	0.793 (0.788, 0.797)		1170 (92.4)	88 (7.6)	
>7 times/week	0.788 (0.785, 0.791)		2146 (91.5)	192 (8.5)	
Coffee intake frequency		0.005			0.009
Never	0.785 (0.782, 0.788)		3814 (90.7)	396 (9.3)	
≤4 times/week	0.786 (0.784, 0.789)		3031 (90.4)	349 (9.6)	
5–7 times/week	0.781 (0.777, 0.784)		3152 (88.2)	410 (11.8)	
>7 times/week	0.781 (0.778, 0.783)		4227 (88.8)	582 (11.2)	
Green tea intake frequency		<0.001			<0.001
Never	0.779 (0.777, 0.781)		7901 (88.1)	1082 (11.9)	
≤4 times/week	0.787 (0.785, 0.790)		4829 (91.1)	501 (8.9)	
5–7 times/week	0.792 (0.786, 0.797)		1100 (91.5)	113 (8.5)	
>7 times/week	0.793 (0.786, 0.800)		394 (93.3)	41 (6.7)	

^1^ Student *t*-test and Wald F-test were used to evaluate the differences in the arithmetic mean between the groups. Rao–Scott chi-square test was used to compare the baseline characteristics across the categorical variables. ^2^ Except the number of participants, all values are weighted. ^3^ CI, confidence interval.

**Table 3:** The results of multiple linear regression analysis for the effects of beverage intake and smoking status on FEV_1_/FVC.

Variables	Crude β (95% CI ^2^)	Fully Adjusted β ^1^ (95% CI ^2^)
**Soda Intake Frequency**		
Per 10 times increasing of monthly intake	0.0011 (0.0006, 0.0016)	−0.0008 (−0.0012, −0.0003)
Never	Ref.	Ref.
≤4 times/week	0.0070 (0.0036, 0.0103)	0.0013 (−0.0017, 0.0043)
4–7 times/week	0.0147 (0.0092, 0.0201)	−0.0016 (−0.0065, 0.0033)
>7 times/week	0.0101 (0.0062, 0.0141)	−0.0052 (−0.0092, −0.0013)
*p* for trend	<0.001	0.016
**Coffee Intake Frequency**		
Per 10 times increasing of monthly intake	−0.0006 (−0.0011, −0.0002)	−0.0006 (−0.0010, −0.0001)
Never	Ref.	Ref.
≤4 times/week	0.0013 (−0.0025, 0.0051)	0.0021 (−0.0013, 0.0056)
4–7 times/week	−0.0045 (−0.0087, −0.0002)	−0.0013 (−0.0052, 0.0027)
>7 times/week	−0.0045 (−0.0082, −0.0007)	−0.0042 (−0.0079, −0.0005)
*p* for trend	0.003	0.031
**Green Tea Intake Frequency**		
Per 10 times increasing of monthly intake	0.0022 (0.0013, 0.0031)	0.0009 (0.0007, 0.0017)
Never	Ref.	Ref.
≤4 times/week	0.0086 (0.0057, 0.0115)	−0.0003 (−0.0029, 0.0023)
4–7 times/week	0.0131 (0.0073, 0.0189)	0.0047 (−0.0006, 0.0010)
>7 times/week	0.0146 (0.0075, 0.0217)	0.0075 (0.0006, 0.0143)
*p* for trend	<0.001	0.029
**Smoking Status**		
Never	Ref.	Ref.
Past-smoker	−0.0356 (−0.0391, −0.0320)	−0.0126 (−0.0170, −0.0082)
Current-smoker	−0.0373 (−0.0417, −0.0330)	−0.0222 (−0.0273, −0.0171)

^1^ Fully adjusted model was adjusted for sex, age, monthly income, education level, drinking status, smoking status, beverage intake, and BMI. ^2^ CI, confidence interval.

**Table 4:** Odds ratios (ORs) for COPD according to beverages intake and smoking status.

Variables	Crude OR (95% CI ^2^)	Fully Adjusted OR ^1^ (95% CI ^2^)
**Soda Intake Frequency**		
Per 10 times increasing of monthly intake	0.96 (0.93, 0.98)	1.04 (1.01, 1.07)
Never	Ref.	Ref.
≤4 times/week	0.79 (0.69, 0.91)	0.94 (0.80, 1.10)
4–7 times/week	0.60 (0.45, 0.78)	1.20 (0.90, 1.60)
>7 times/week	0.67 (0.56, 0.82)	1.30 (1.04, 1.64)
*p* for trend	<0.001	0.041
**Coffee Intake Frequency**		
Per 10 times increasing of monthly intake	1.02 (1.00, 1.04)	1.01 (0.98, 1.03)
Never	Ref.	Ref.
≤4 times/week	1.04 (0.86, 1.25)	1.02 (0.83, 1.26)
4–7 times/week	1.30 (1.08, 1.56)	1.10 (0.90, 1.34)
>7 times/week	1.22 (1.04, 1.45)	1.12 (0.92, 1.37)
*p* for trend	0.004	0.467
**Green Tea Intake Frequency**		
Per 10 times increasing of monthly intake	0.92 (0.87, 0.97)	0.98 (0.93, 1.03)
Never	Ref.	Ref.
≤4 times/week	0.73 (0.63, 0.84)	1.02 (0.87, 1.19)
4–7 times/week	0.69 (0.52, 0.90)	0.97 (0.73, 1.30)
>7 times/week	0.53 (0.35, 0.80)	0.67 (0.42, 1.08)
*p* for trend	<0.001	0.331
**Smoking Status**		
Never	Ref.	Ref.
Past-smoker	3.59 (3.09, 4.18)	1.78 (1.42, 2.24)
Current-smoker	3.80 (3.25, 4.43)	2.58 (2.06, 3.24)

^1^ Fully adjusted model was adjusted for sex, age, monthly income, education level, drinking status, smoking status, beverage intake, and BMI. ^2^ CI, confidence interval.

**Table 5:** ORs (95% confidence interval) for COPD by joint effect between the frequency of soda intake and pack-year smoking.

Variables	Low Pack-Year	High Pack-Year	Pack-Year within Strata of Soda Intake
Low soda intake	1.00 (reference)	2.04 (1.68, 2.48)	2.04 (1.68, 2.48)
High soda intake	1.26 (1.02, 1.55)	3.00 (2.32, 3.89)	1.96 (1.38, 2.79)
Soda intake within strata of pack-year	1.26 (1.02, 1.55)	1.41 (1.05, 1.89)	

Measurement of interaction on additive scale: RERI = 0.71 (−0.02 to 1.44); *p* = 0.058; Measurement of interaction on multiplicative scale: ratio of ORs = 1.17 (0.86 to 1.60); *p* = 0.314; Models were adjusted for age, sex, monthly income, education levels, drinking status, coffee intake frequency, green tea intake frequency, and BMI.

**Table 6:** ORs (95% confidence interval) for COPD by joint effect between the frequency of coffee intake and pack-year smoking.

Variables	Low Pack-Year	High Pack-Year	Pack-Year within Strata of Coffee Intake
Low coffee intake	1.00 (reference)	2.34 (1.78, 3.08)	2.34 (1.78, 3.08)
High coffee intake	1.11 (0.93, 1.33)	2.28 (1.84, 2.82)	1.60 (1.28, 2.00)
Coffee intake within strata of pack-year	1.11 (0.93, 1.33)	1.05 (0.78, 1.41)	

Measurement of interaction on additive scale: RERI = −0.17 (−0.82 to 0.49); *p* = 1.385; Measurement of interaction on multiplicative scale: ratio of ORs = 0.88(0.64 to 1.21); *p* = 0.430; Models were adjusted for age, sex, monthly income, education levels, drinking status, soda intake frequency, green tea intake frequency, and BMI.

**Table 7:** ORs (95% confidence interval) for COPD by joint effect between the frequency of green tea intake and pack-year smoking.

Variables	Low Pack-Year	High Pack-Year	Pack-Year within Strata of Green Tea Intake
Low green tea intake	1.00 (reference)	2.20 (1.83, 2.64)	2.20 (1.83, 2.64)
High green tea intake	1.07 (0.85, 1.35)	2.03 (1.49, 2.76)	1.92 (1.21, 3.05)
Green tea intake within strata of pack-year	1.07 (0.85, 1.35)	0.97 (0.70, 1.33)	

Measurement of interaction on additive scale: RERI = −0.24 (−0.92 to 0.44); *p* = 1.516; Measurement of interaction on multiplicative scale: ratio of ORs = 0.86 (0.59 to 1.26); *p* = 0.441; Models were adjusted for age, sex, monthly income, education levels, drinking status, soda intake frequency, coffee intake frequency, and BMI.

## Supplementary Materials

The following are available online at https://www.mdpi.com/1660-4601/17/7/2611/s1, Table S1: ORs for COPD according to beverages intake and smoking status in a sensitivity analysis.
