# Supplementary material for: The Joint Effects of Some Beverages Intake and Smoking on Chronic Obstructive Pulmonary Disease in Korean Adults: Data Analysis of the Korea National Health and Nutrition Examination Survey (KNHANES), 2008–2015
Source: Int J Environ Res Public Health. 2020 Apr 10;17(7):2611. doi: 10.3390/ijerph17072611 (PMC7178176; doi:10.3390/ijerph17072611)
Supplement: Supplementary file 1 [file ijerph-17-02611-s001.zip › ijerph-744222-supplementary.pptx]

## Slide 1
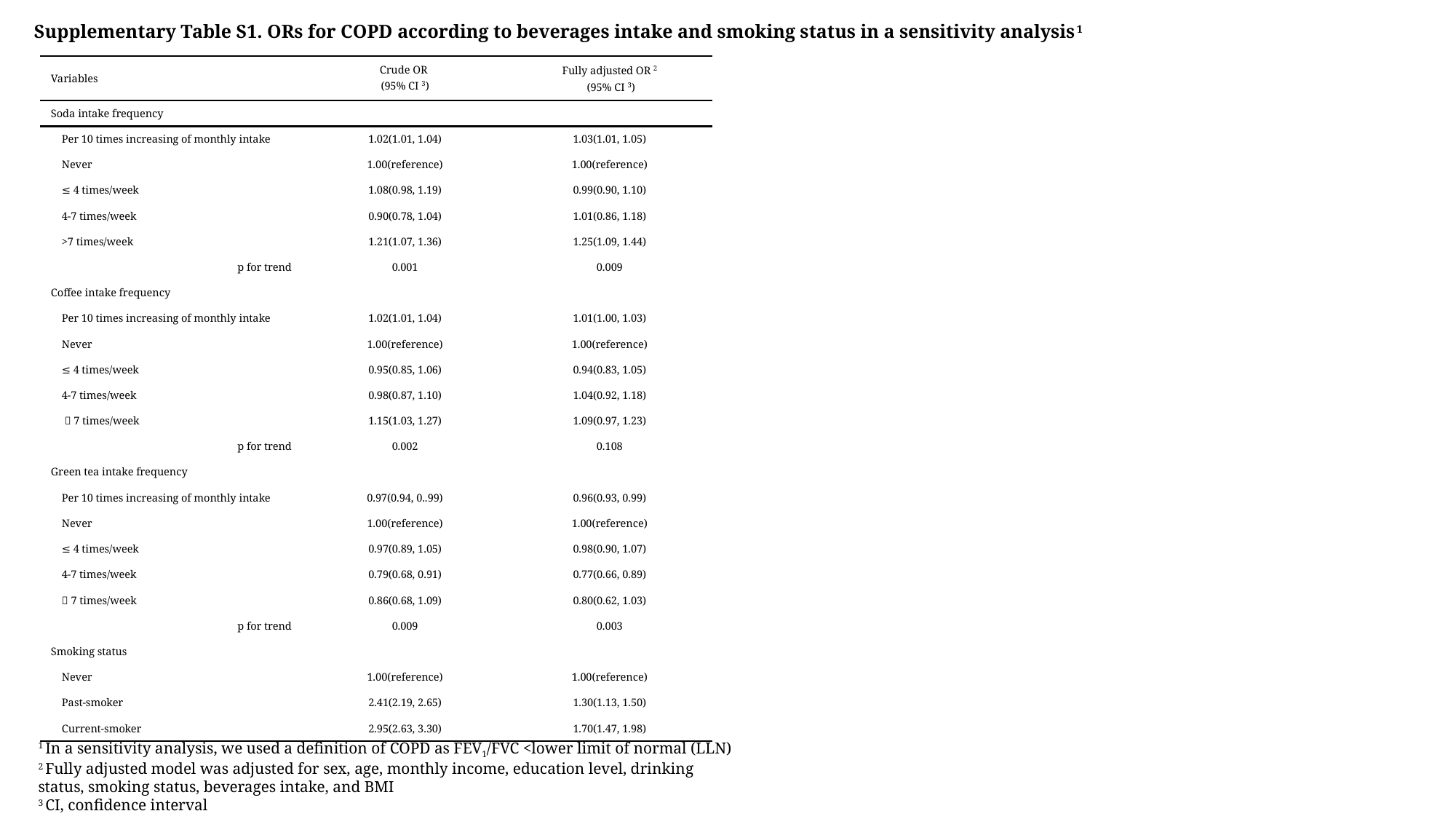

Supplementary Table S1. ORs for COPD according to beverages intake and smoking status in a sensitivity analysis1
| Variables | Crude OR (95% CI 3) | Fully adjusted OR 2 (95% CI 3) |
| --- | --- | --- |
| Soda intake frequency | | |
| Per 10 times increasing of monthly intake | 1.02(1.01, 1.04) | 1.03(1.01, 1.05) |
| Never | 1.00(reference) | 1.00(reference) |
| ≤ 4 times/week | 1.08(0.98, 1.19) | 0.99(0.90, 1.10) |
| 4-7 times/week | 0.90(0.78, 1.04) | 1.01(0.86, 1.18) |
| >7 times/week | 1.21(1.07, 1.36) | 1.25(1.09, 1.44) |
| p for trend | 0.001 | 0.009 |
| Coffee intake frequency | | |
| Per 10 times increasing of monthly intake | 1.02(1.01, 1.04) | 1.01(1.00, 1.03) |
| Never | 1.00(reference) | 1.00(reference) |
| ≤ 4 times/week | 0.95(0.85, 1.06) | 0.94(0.83, 1.05) |
| 4-7 times/week | 0.98(0.87, 1.10) | 1.04(0.92, 1.18) |
| ＞7 times/week | 1.15(1.03, 1.27) | 1.09(0.97, 1.23) |
| p for trend | 0.002 | 0.108 |
| Green tea intake frequency | | |
| Per 10 times increasing of monthly intake | 0.97(0.94, 0..99) | 0.96(0.93, 0.99) |
| Never | 1.00(reference) | 1.00(reference) |
| ≤ 4 times/week | 0.97(0.89, 1.05) | 0.98(0.90, 1.07) |
| 4-7 times/week | 0.79(0.68, 0.91) | 0.77(0.66, 0.89) |
| ＞7 times/week | 0.86(0.68, 1.09) | 0.80(0.62, 1.03) |
| p for trend | 0.009 | 0.003 |
| Smoking status | | |
| Never | 1.00(reference) | 1.00(reference) |
| Past-smoker | 2.41(2.19, 2.65) | 1.30(1.13, 1.50) |
| Current-smoker | 2.95(2.63, 3.30) | 1.70(1.47, 1.98) |
1 In a sensitivity analysis, we used a definition of COPD as FEV1/FVC <lower limit of normal (LLN)
2 Fully adjusted model was adjusted for sex, age, monthly income, education level, drinking status, smoking status, beverages intake, and BMI
3 CI, confidence interval
